# Expert Opinion on Three Phage Therapy Related Topics: Bacterial Phage Resistance, Phage Training and Prophages in Bacterial Production Strains

**DOI:** 10.3390/v10040178

**Published:** 2018-04-05

**Authors:** Christine Rohde, Grégory Resch, Jean-Paul Pirnay, Bob G. Blasdel, Laurent Debarbieux, Daniel Gelman, Andrzej Górski, Ronen Hazan, Isabelle Huys, Elene Kakabadze, Małgorzata Łobocka, Alice Maestri, Gabriel Magno de Freitas Almeida, Khatuna Makalatia, Danish J. Malik, Ivana Mašlaňová, Maia Merabishvili, Roman Pantucek, Thomas Rose, Dana Štveráková, Hilde Van Raemdonck, Gilbert Verbeken, Nina Chanishvili

**Affiliations:** 1 Department of Microorganisms, Leibniz Institute DSMZ—German Collection of Microorganisms and Cell Cultures, 38100 Braunschweig, Germany; Christine.Rohde@dsmz.de; 2 Department of Fundamental Microbiology, University of Lausanne, 1015 Lausanne, Switzerland; Gregory.Resch@unil.ch; 3 Laboratory for Molecular and Cellular Technology, Queen Astrid Military Hospital, 1120 Brussels, Belgium; jean-paul.pirnay@mil.be (J.-P.P.); maya.merabishvili@pha.ge (M.M.); thomas.rose@mil.be (T.R.); hilde.vanraemdonck@mil.be (H.V.R.); Gilbert.Verbeken@mil.be (G.V.); 4 Laboratory of Gene Technology, Department of Biosystems, 3000 Leuven, Belgium; blasdelb@gmail.com; 5 Department of Microbiology, Institut Pasteur, 75015 Paris, France; laurent.debarbieux@pasteur.fr; 6 Faculty of Dental Medicine, The Hebrew University of Jerusalem, Jerusalem 9112001, Israel; daniel.gelman@mail.huji.ac.il (D.G.); ronenh@ekmd.huji.ac.il (R.H.); 7 Bacteriophage Laboratory, Hirszfeld Institute of Immunology and Experimental Therapy, Polish Academy of Sciences, 53-114 Wroclaw, Poland; agorski@ikp.pl; 8 Department of Clinical Immunology, Transplantation Institute, Medical University of Warsaw, 02-006 Warsaw, Poland; 9 Department of Pharmaceutical and Pharmacological Sciences, KU Leuven, 3000 Leuven, Belgium; isabelle.huys@kuleuven.be; 10 Eliava Institute of Bacteriophage, Microbiology and Virology, Gotua Street 3, 0160 Tbilisi, Georgia; elene.kakabadze@pha.ge (E.K.); khatuna.makalatia@pha.ge (K.M.); 11 Institute of Biochemistry and Biophysics, Polish Academy of Sciences, 00-901 Warsaw, Poland; lobocka@ibb.waw.pl; 12 Autonomous Department of Microorganisms’ Biology, Faculty of Agriculture and Biology, Warsaw University of Life Sciences—SGGW, 02-787 Warsaw, Poland; 13 University of Turin, 10124 Turin, Italy; alice.maestri@edu.unito.it; 14 Centre of Excellence in Biological Interactions, Department of Biological and Environmental Science, Nanoscience Center, University of Jyväskylä, Survontie 9C, FI-40014 Jyväskylä, Finland; gabriel.m.almeida@jyu.fi; 15 Chemical Engineering Department, Loughborough University, Leicestershire LE11 3TU, UK; d.j.malik@lboro.ac.uk; 16 Department of Experimental Biology, Faculty of Science, Masaryk University, 60000 Brno, Czech Republic; iva.maslanova@gmail.com (I.M.); pantucek@sci.muni.cz (R.P.); stverakova@mbph.cz (D.Š.); 17 Laboratory for Bacteriology Research, Faculty Medicine & Health Sciences, Ghent University, 9000 Ghent, Belgium; 18 MB Pharma, 120 00 Prague 2 Vinohrady, Czech Republic

**Keywords:** Bacteriophage, phage therapy, resistance, adaptation, prophage, production, regulation

## Abstract

Phage therapy is increasingly put forward as a “new” potential tool in the fight against antibiotic resistant infections. During the “Centennial Celebration of Bacteriophage Research” conference in Tbilisi, Georgia on 26–29 June 2017, an international group of phage researchers committed to elaborate an expert opinion on three contentious phage therapy related issues that are hampering clinical progress in the field of phage therapy. This paper explores and discusses bacterial phage resistance, phage training and the presence of prophages in bacterial production strains while reviewing relevant research findings and experiences. Our purpose is to inform phage therapy stakeholders such as policy makers, officials of the competent authorities for medicines, phage researchers and phage producers, and members of the pharmaceutical industry. This brief also points out potential avenues for future phage therapy research and development as it specifically addresses those overarching questions that currently call for attention whenever phages go into purification processes for application.

## 1. Foreword

This article is a reflection of three roundtable discussions in question–answer format held at the “Centennial Celebration of Bacteriophage Research” conference, which took place in Tbilisi, Georgia, on 26–29 June 2017. The goal was to elaborate a concerted expert opinion, based on clinical experience and scientific knowledge, on three commonly identified knowledge gaps with regard to phage therapy and the manufacturing of adequate phage therapy products: bacterial phage resistance, phage training and the presence of prophages in bacterial production strains. Whenever phages are foreseen for application, they need to undergo a careful pre-selection after intensive application-oriented biological investigation; only such phages should go into a purification process chain. However, the combination of single phages into cocktails creates additional more complex investigation. The goal of this brief is to inform phage therapy stakeholders from the academic, industrial, medical and regulatory areas on these three contentious issues in the context of an increasing demand for human and veterinary phage applications.

## 2. Bacterial Resistance to Phages

The antagonistic co-evolution between bacterial hosts and their infecting phages is considered to be an important driver of ecological and evolutionary processes in microbial communities [1]. In the light of a renewed interest in using phages to treat bacterial infections, in vitro studies indicate that bacteria–phage co-evolution could be an important factor in the success (or failure) of certain phage therapy applications. The evolution of bacterial resistance to individual phages is often (if not always) observed in vitro, but with considerable variance. However, phages have evolved multiple strategies to overcome the antiviral mechanisms they encounter when infecting bacterial cells [2], such as anti-CRISPR (Clustered Regularly Interspaced Short Palindromic Repeats) proteins [3]. On the other hand, BREX and DISARM are phage resistance systems widespread in bacterial genomes that have been recently discovered [4,5].

In experimental settings, phage-resistant bacteria are observed to emerge rapidly, but often at significant fitness costs, commonly including a reduced growth rate in the absence of phages [6]. Evolved (pre-adapted or “trained”) phages were shown to be more effective in reducing the densities of chronic bacterial isolates [7]. The in vivo evolution of bacterial resistance to phages in human clinical practice seems inevitable, but this has been poorly documented in the scientific literature to date. When rats with aortic experimental endocarditis (EE) were treated with an anti-*Pseudomonas* phage cocktail, phage-resistant mutants with impaired infectivity were shown to emerge in vitro but not in vivo, presumably because resistance mutations in bacteria involved bacterial surface determinants necessary for infectivity (e.g., genes involved in pilus motility and lipopolysaccharide (LPS) formation) [8]. In a recent long-term study that followed co-evolution between phages and bacteria in a natural environment, *Flavobacterium columnare* isolates were found to be generally resistant to phages from the past and susceptible to phages isolated in years after bacterial isolation. Bacterial resistance had selected for increased phage infectivity and host range. Bacterial resistance was correlated to the appearance of new anti-phage spacers in CRISPR loci, and on several occasions the corresponding protospacer regions in the genome of phages isolated in the following samplings were found to be modified in response. This study shows that, in natural conditions (e.g., natural phage/bacteria ratios and diversities), phages and bacteria co-evolve in a continuous arms race [9].

One expert further notes that the in vivo growth rates as well as the metabolic status of hosts in a polymicrobial biofilm may typically be quite different compared to observations in in vitro studies where the host is in the log growth phase in a nutrient rich environment.

### 2.1. Strategies to Minimize Bacterial Phage Resistance

Most of the round table participants had no idea of the frequency of emergence of bacterial phage resistance in clinical practice. According to one group’s experience, however, the number of patients in whom a pathogen acquired resistance to the phage used during therapy may vary from 17% (*Staphylococcus aureus* phages) to 85% (*Escherichia coli* phages) [10]. The majority of the participants feel that it is difficult to develop a phage cocktail to which bacteria would not be able to evolve resistance during therapy. In contrast, three participants, including two biopharmaceutical researchers, presume that it might, however, be possible to develop resistance-proof therapeutic phage cocktails, using phages with a broad host range and targeting highly conserved structures that are essential for bacterial survival and/or infectivity. Two of them note that the phage resistance problem is not caused by the de novo emergence of phage resistant clones, but by the selection of naturally present phage resistant isolates harboring antiviral mechanisms such as restriction modification systems and CRISPR/Cas (CRISPR associated proteins) systems. It is not hard to imagine that the spread of these mechanisms through horizontal gene transfer may indeed be the main driver of bacterial phage resistance occurrence in natural environments, with large population diversities and dynamics, but little is known if this is also the case in the patient’s infection site. These phage-resistance-proof cocktails would need to be updated regularly to target newly selected phage resistant clones. One expert stresses that, in the experience at the Eliava Phage Therapy Center, even when a phage (cocktail) shows no in vitro lytic activity against an infecting bacterial strain (e.g., using the spot test), this phage (cocktail) might still be clinically effective in vivo. A reason for this might be that these phage resistant bacteria display an impaired virulence to support an ongoing infection and may be more easily managed by the immune system [8].

All participants do believe that it is possible to minimize the occurrence of bacterial phage resistance. Phages should be selected that belong to different families/groups and that individually show important infectious ability, such as a broad host range, high efficiency of plating (EOP), high adsorption rates, short latent periods, large burst sizes and a low inclination to select resistance (e.g., as determined by the Appelmans method [11]), and which act synergistically when mixed into one cocktail. Ideally, phage cocktails should be composed of phages that adsorb to different highly conserved bacterial cell wall structures or virulence factors and exert a selective pressure on different antiviral resistance mechanisms in the same target bacterium. Based on an extensive experience as phage researcher in the Eliava Institute, one expert stresses that there is a limit to the number of phages that can be successfully combined into a single phage cocktail, as some phages are bound to be incompatible or to compete for the same bacterial host. In addition, some experts feel that these cocktails might need to be tailored to a specific patient and when necessary adapted in vitro during therapy to reduce the risk of generating persistent bacterial phage resistance. Knowing which bacterial structures phages interact with, as well as a better understanding of the resistance mechanisms they elicit, are crucial for the success of this approach. One participant states that bacterial resistance against any phage cocktail will inevitably occur at some point during treatment, but that by then the amount of pathogenic bacteria might have been sufficiently reduced (the equilibrium is restored) for the patient’s immune system (or other antibacterials) to resolve the infection. It was suggested earlier that synergy between phages and the patient’s immune system might be required for the resolution of the bacterial disease in certain indications [12].

Should sequential strategies in which individual active phages are applied one after the other, so that treatment does not simultaneously select for broad resistance in the targeted bacteria, be considered [6]? Most participants believe that this approach could be (more) effective, but would be very difficult to implement in clinical practice. Especially in severe acute infections, this approach would require large collections of different lytic phage clones and rapid (automated) phage selection and adaptation techniques. Rapid sequencing technologies and algorithm based phage selection of phages from libraries may allow rational selection of phage cocktails targeting different conserved receptors. In addition, sequential strategies are only feasible for hospitalized patients or ambulant patients visiting the hospital on a regular basis (e.g., every day). It would also require the use of significantly more diagnostic tools than is currently the habit of medical doctors and veterinarians when administering broad-spectrum antibacterials. One expert notes that sequential approaches could also be achieved using burst release and time delayed release systems [13]. Another participant fears that the sequential approach will give bacteria the opportunity to develop resistance against one active phage at the time. Two participants assume that decreases of phage efficiency are partly due to the patient’s immune response and that sequential approaches could therefore be more effective.

In vitro studies indicate that pre-adapting lytic phages to a pathogen leads to increased pathogen clearance and lowered resistance evolution [7], but will it lower the occurrence of bacterial phage resistance in clinical practice? Most participants presume that it may, as properly pre-adapted phages could harbor mutations (e.g., single-nucleotide polymorphisms (SNPs) or short deletions), which would allow them to escape antiviral mechanisms, but they would like to see clinical evidence of this. In addition, pre-adaptation could also result in phages with broader host ranges and increased infectious abilities (see Section 3). Two participants claim that pre-adapting phages will likely only result in a faster co-evolution process and will have no impact on bacterial phage resistance.

One participant points out that phage resistance is sometimes due to an interruption of the lytic cycle and therefore advocates the use of phage endolysins. Some experts propose to combine the use of phage cocktails and antibiotics, while choosing phages that interact with relevant antibiotic resistance determinants. As such, bacterial phage resistance could lead to (regained) increased susceptibility to antibiotics [14], leading to synergistic selective pressure. More studies are needed to investigate the significance of this synergistic effect in vivo.

### 2.2. Phages in Agriculture, Fisheries and Food

Phage products are on the market for the decontamination of food pathogens and phage probiotics as well as products for farms are in development [15]. However, what do we know about the impact of the large-scale and empirical use of phages in agriculture and food on bacterial resistance to phages or on the shape and diversity of bacterial populations in the environment or in field trials?

Most participants believe that, in a way analogous to antibiotics, the uncontrolled widespread use of phages in agriculture and food decontamination might become a contributor to phage resistant bacterial diseases if phage therapy is to be (re-)integrated in human medicine. Some participants fear that bacterial phage resistance determinants will spread (e.g., through horizontal gene transfer should this transfer pathway definitely play a major role) and persist in the environment. In addition, since complex interactions between phages and bacteria already play significant roles in the composition of environmental microbial communities (e.g., bacterial adaptation to stress via phage transduction), there could be important and unpredictable impacts on the ecosystem.

One expert in aquaculture-associated phage research suggests that phage resistant bacteria might not be able to persist in the environment in the absence of the applied phage, due to the fitness cost of typical mutations that confer phage resistance. It is, however, not clear how phage resistance would differ from antibiotic resistance in this perspective. Three participants argue that the use of phages in agriculture, fisheries and food will likely increase bacterial phage resistance, but they are confident that the host–parasite co-evolutionary arms race will always result in the emergence of successful phages [16]. One expert points out that many bacterial serotypes that cause cattle/fish/plant diseases are different from those causing human disease, but that this does not exclude the emergence of cross-resistance.

With the exception of two participants, who do not believe that bacterial phage resistance will persist in the environment, most experts suggest restricting the use of phages to a greater or lesser extent to prophylactically limit the potential spread of bacterial phage resistance in anticipation of relevant data. The majority proposes avoiding the empirical (without previous diagnosis) use of phages and to control and limit the scale of phage applications especially in agriculture, fisheries and food. A few participants would like to reserve the use of phages for serious (antibiotic resistant) human infections at first priority, with phages obtained only upon medical prescription. Finally, all participants feel that phage products should not be produced, marketed and used as if they were a new class of antibiotics and suggest that phage therapy should have its own regulatory platform, allowing flexible approaches including the timely production, composition, and adaptation of phage products. Two experts stress that it is important that the production of phage preparations should comply with Good Manufacturing Practices (GMP). To summarize, more fundamental research is required in order to differentiate between bacterial phage resistance mechanisms, between in vitro and in vivo resistance phenomena and to quantify these more precisely. This is necessary to better understand therapeutic phage efficacy. Finally, and in the context of the patient’s immune response, such comparative data assessments will shed light on the truth of bacterial phage resistance.

## 3. Phage Training

### 3.1. Phage Therapy and the Problem of Heterogeneity in Bacterial Populations

It is well known that bacterial populations are heterogeneous and that this heterogeneity can originate either genetically or phenotypically [17,18,19]. Heterogeneity is already considered problematic from a therapeutic point of view when it concerns bacterial susceptibility to antibiotics [20,21,22]. Similarly, it could challenge phage therapy since it is known that phage-resistant variants pre-exist within bacterial populations and can be relatively easily selected in vitro through bet hedging [8,17,23]. A very recent case of a patient suffering from an *Acinetobacter baumannii* disseminated infection treated with phage therapy highlighted the clinical relevance of such phage-resistant variants [24]. Indeed, phage-resistant clones were selected in the patient during the treatment course, which necessitated adjustment of the phage cocktail composition twice. In this case, it stands to the credit of the involved teams that they were able to sequentially produce tailored cocktails of natural phages (i.e., non-trained) able to kill the resistant clones, within the very limited amount of time available to the patient.

### 3.2. Phage Training?

In parallel to the adaptation of the bacterial host to the attacking phage, phages in turn naturally adapt to their hosts during co-evolution in common habitats following an arms race or fluctuating selection processes [25,26,27], explaining why both bacteria (the prey) and phages (the predator) are still present on the surface of our planet after billions of years of co-habitation. As a result, phages that have been evolved to better fit the context of phage therapy can be selected for in vitro and in vivo. Various forms of phage training, also known as phage adaptation or phage pre-adaptation, have been developed to select for these evolved phages through experimental procedures performed in a laboratory. It is generally acknowledged that phage training protocols originated from the so-called Appelmans experiment reported in 1921 [11]. At first, Appelmans designed his classical eponymous experiment to titer a phage solution more precisely than d’Hérelle performed at that time. In order to do so, Appelmans was inspired by an approach of serial dilutions to quantify bacteria in water samples as described in chapter III of Miquel’s “*Manuel Pratique d’analyse bactériologique des eaux*” published in 1891. The principle is relatively simple and is still used today with some modifications.

In this original study, Appelmans exposed a liquid culture of a susceptible bacterium to serial dilutions of the phage (up to 10^−12^), an experiment very similar to what is currently done in the macro-dilution method for the determination of the Minimum Inhibitory Concentration (MIC) of antibiotics. After incubation (incubation time is only indicated in Appelmans’ paper as “immediate” or “lately”), the tubes in which bacteria were able to grow were considered to be devoid of phages and the tubes in which no growth was observed were considered as containing phages. Taking into consideration the dilution factor, Appelmans was able to precisely determine the phage titer in the undiluted solution. This experiment also allowed him to further validate d’Hérelle’s hypothesis about the nature of phage amplification on bacteria. Moreover, Appelmans decided to perform an additional series of experiments in which he serially diluted the phage into 50% alcohol or 5% phenol. Indeed, at that time it had already been published that the phage Appelmans used in his study was stable when exposed to both agents. However, stability was only tested in a highly concentrated phage solution. Surprisingly, Appelmans, with his dilution approach, highlighted the fact that not all phages in the solution were equally resistant to both agents. Indeed, while the non-exposed phage solution was still active at a 10^−10^ dilution, the corresponding phage solution exposed to 50% alcohol remained active only when diluted up to 10^−6^ independent of the incubation time (6 h, 24 h, 3 days, 10 days or 20 days). An additional dilution of this solution led to its inactivity, arguing for the presence of a fixed number of phages insensitive to the agent in the test tube. Appelmans made the same observation with 5% phenol except that the number of phages able to resist this treatment was much lower (dilution 10^−3^ still active). In other words, this experiment demonstrated selection through dilution of phage variants able to resist to some chemicals otherwise toxic for the majority of individuals in the phage population. This is indeed exactly the principle of phage training in which phage variants able to very efficiently lyse a bacterial population are selected through dilution. There are indications that Félix d’Herelle introduced the concept of serial passages that were not performed in the original Appelmans experiments [28].

Two primary protocols of phage training for expanded host range have been reported in the literature [7,29,30]. Firstly, a phage/bacteria mixture is simply diluted into fresh growth medium after a period of co-incubation [7,30]. In the second, a fixed concentration of bacteria is co-incubated with serial dilution of a phage stock in growth medium for 16–24 h. The next morning, the mixture in the tube in which lysis occurred at the lowest phage concentration is further chloroformed and filtered before being serially re-diluted and mixed with sample of a fresh culture of the ancestor bacteria [31]. In both protocols the procedure can be repeated for several “passages”.

### 3.3. Outcome of Phage Training

Experts pointed out that while several mechanisms of phage adaptation ensuring phage propagation on co-evolving hosts were previously described [32]; it is only more recently that the benefits of experimental phage training started to be investigated in controlled assays. In a first study [33], the training *P. aeruginosa* phage PAK_P3, which initially showed only slight lysis on strain CHA, led to the selection of phage P3-CHA with significantly increased in vitro efficiency of plating. This in vitro result was confirmed in vivo in a mouse model of lung infection with 100% versus 20% survival rate achieved by P3-CHA and PAK_P3, respectively. Strikingly, this improved in vivo activity was reported to be due to only two single nucleotide changes in different putative open reading frames (ORFs). This result highlights how quickly a new therapeutic phage with tremendously increased infectivity can emerge in a natural co-evolution process, and could therefore be artificially selected for by phage training. In another study it has been shown that four evolved *P. aeruginosa* phages obtained within two passages over four days were more efficient than ancestral phages in reducing in vitro mean bacterial densities of ten *P. aeruginosa* strains [7]. Accordingly, *P. aeruginosa* phages LKD16 and 14/1 pre-adapted over six serial passages were shown to target clones within a bacterial population of the strain PAO1 that were originally resistant to the ancestor phages. Indeed, the proportion of susceptibility over 20 different clones increased from 80% to 85% for the ancestral phages to 100% for the evolved phages [26]. Therefore, increased infectivity of evolved over ancestral phages is usually attributed to a decreased capacity of the ancestral bacterial strains to evolve resistance towards the evolved phages.

### 3.4. Relevance and Implementation of Phage Training in the Clinic

While one expert described how phage training has been common practice for more than 80 years at the Eliava Institute, all tend to think that there is no doubt that relying on evolved phages able to sidestep bacterial heterogeneity would have been highly desirable for the case discussed above (see Section 3.1) and would therefore be relevant in the clinic in general. Although a pre-clinical study has reported the benefit of a trained phage relative to its original counterpart in vivo, comparable studies should be set up to shed additional light on this biological phenomenon [33].

The implementation of phage training in clinical protocols is appealing for at least two reasons. First, having access to phages covering 100% of the clones within the population of a given strain could dramatically increase the success of phage therapy in a given patient. For instance, *P. aeruginosa* populations in the lungs of patients with cystic fibrosis (CF) harbor a very high phenotypic diversity [34]. Therefore, training for phages that would render them able to cover this phenotypic diversity in CF patients could be clinically significant. Secondly, having access to single phages covering close to 100% of the circulating strains of a given pathogen would allow usage of a very limited number of phages (or, in rare cases, even a single phage) for many patients, thus simplifying the production process.

All experts further distinguished two situations, i.e., acute and chronic infections. If phage training is demonstrated in the future to be an efficient way to significantly improve therapeutic outcomes, several experts noticed that implementation in chronic situations where time is not such an issue would in principle be considerably easier. However, the addition of phage training steps to a treatment protocol would be time consuming and feasible only if sufficient qualified personnel were available. In such a situation, experts pointed out that phage training could be done by either following (i) a one-size-fits-all strategy by training phages on already available representatives of local strains, which will help with setting up and regularly updating specialized phage collections, or (ii) a tailored strategy by training phages on the patient’s strain as soon as it became available in a form of highly personalized medicine. Of note, this latter strategy is applied at the Eliava Institute in the process of development of so-called “autophages”.

Accordingly, many experts agreed that in intensive care units (ICUs), where patients need to be treated within minutes or hours, implementation of a tailored strategy would be difficult due to time limitations. Indeed, as discussed before, phage training protocols usually require a week to be performed or at minimum 24–48 h in case of a single passage [29]. Nevertheless, an expert pointed out that often the strain that will cause the life-threatening condition, often including sepsis, in ICU patients is known days before as the dominant colonizing strain. In such a situation, patients could be decolonized with available phages (see above) and autophages could then be developed to adjust the treatment and cover potential phage-resistant variants selected by the former phages, in a way that is similar to what occurred in the case discussed in Section 3.1). This strategy is very similar to the situation where patients are first treated with broad-spectrum antibiotics and treatment is adjusted later according to an antibiogram. However, in acute situations where the strain would not be available in advance, experts agree that the emergency use of broad-host range phage cocktails could be a viable strategy. As a conclusion, while all experts rather agree on the clinical importance of developing trained phages, some think that detailed pre-clinical and clinical studies still need to be performed to properly evaluate “cost vs. benefits” and decide if it is worthwhile to invest into the time- and resources-consuming development of trained phage collections. If such a strategy were shown to be a viable option, phage training could either be implemented as a one-size-fits-all or a tailored strategy depending on the patient’s condition.

### 3.5. Regulatory Considerations Regarding Trained Phages

In answer to the question “should trained phages be considered as natural phages?” if one would use them in clinical trials, a large majority of experts answered “yes”. Indeed, phage training is based on a naturally occurring event and does not rely on “human-guided” modification of the phage genome through for instance synthetic biology or any other tools. A trained phage is a phage in which random mutations have been introduced by co-evolution with bacteria as it happens in nature. In other words, a trained phage is a phage variant that pre-exists in nature and is selected and amplified in the laboratory through natural, but accelerated, co-evolution. Since adapted phages are variants selected from a population of natural phages, their status regarding regulatory agencies should be similar to the original phage.

## 4. Prophages in Bacterial Production Strains

### 4.1. Relevance of Prophages to the Production of Therapeutic Phages

Temperate phages are champions of evolution, but unwanted during pharmaceutical phage production. Why do we address the “prophage issue” here and discuss it in depth? It is a matter of course to consider all relevant questions and parameters before therapeutic phage preparations are produced. As discussed earlier by international experts, production of phage preparations for medical application must follow defined procedures and quality assessments [35,36,37,38]. It is important to understand the biology of the two different types of phages that are in fact two different forms of life: obligately lytic, virulent phages are attractive potent alternatives to antibacterial drugs as these phages kill their bacterial host cells upon infection. Temperate phages lysogenize their bacterial host cells, exist as prophages after integration into the bacterial chromosomes or as plasmid-like extra-chromosomal elements. When “induced”, they then change their life cycle and behave like virulent phages while lysing bacterial hosts. The term “temperate phage” thus refers to the character of this form of phage life whereas “prophage” describes a status in the complex life cycle of such a phage. Temperate phages exist in most bacteria as prophages, often abundantly, being normal parts of bacterial genomes.

Indeed, prophages are extremely abundant elements in the biosphere. Consequently, they also belong to our own microbiome and are part of its virome, and can be described as a phageome [39]. It has even been found that prophages in our microbiome are communicating via signaling peptides though the “arbitrium code” system [40]. Lysogeny clearly plays an enormous evolutionary role, with temperate phages substantially involved in co-evolution processes of both bacteria and phages [41]. This is relevant also in the context of this article: we are carrying countless prophages. Both, the microbiome and the individual macro-organism represent the holobiont, this rather recent and Solomonic perception of life includes all forms of life that contribute to an individual’s existence and health [42].

Prophage induction occurs when expression of the transcriptional repressor keeping host lethal genes involved in lytic infection shut off is impaired to such an extent that lytic infection begins. Classically, this impairment is known to be caused by stresses such as UV irradiation, mutagens, quorum-sensing signaling molecules, fluoroquinolones, oxidative stresses (such as hydrogen peroxide) or a temperature shift that generates sufficient transient damage to the bacterial cell. In addition, it has also been long known that infection by lytic phages, like happens as an inherent part of the lytic phage production process, also strongly induces the excision of prophages. Indeed, five of seven *P. aeruginosa* phages examined by Blasdel et al. (including lytic phages being currently used for therapy) induce the transcription of at least part of at least one prophage element in their PAO1 host [43]. If prophage induction successfully highjacks the phage infection during production, then the prophage will be released into the medium, and possibly contaminate the therapeutic preparation that should exclusively contain the lytic phage. Finally, the induction of active prophages can also occur spontaneously with various frequencies [44], typically one in 10^3^–10^5^ cells, but sometimes one in 10^2^ cells [45], and certain toxin-encoding phages were shown to be induced spontaneously with higher frequency than their non-toxin-encoding relatives [46]. As a consequence, populations of lysogens are typically contaminated with free temperate phages [47].

According to pharmaceutical standards, such a contamination of therapeutic phages with temperate phages is out of the question. Selection of the strain and a carefully adapted experimental quality control procedure is crucial as both phage types, the therapeutic phage and a temperate contaminant phage, cannot be separated chemically or physically as they share biochemical and structural similarities. Prophages are common in probably all taxonomic groups of bacteria, and knowledge about them is growing as more strains are sequenced and bioinformatics tools are getting more sophisticated. Comparative genomics is a fundamentally important scientific development. Indeed, many prophages are associated with pathogenicity, such as in *E. coli*, *Streptococcus pyogenes*, *Salmonella enterica*, *Staphylococcus aureus* and can encode for exotoxins like in *E. coli* EHEC or *Vibrio cholera* as well as a rather broad spectrum of enzymes significant for bacterial virulence [48].

However, bacterial genomes often bear non-complete (cryptic) prophages that cannot be induced or released from the cell. Due to the intensive relatedness to the human microbiome, most clinically important bacterial isolates, especially those belonging to the ESKAPE group (Enterococci (VRE), *S. aureus* (MRSA), *Klebsiella pneumoniae* (Carbapenem resistant), *A. baumannii*, *P. aeruginosa*, *Enterobacteriaceae* (ESBL)) contain both intact and cryptic prophage sequences in their genomes. It is our own rich and dense microbiome habitat that inevitably causes constant genetic exchange and co-evolution of bacteria and their phages. Especially for clinical isolates, it is a selective advantage to carry many prophages, which often encode genetic cassettes that benefit their hosts such as virulence factors [49,50]. Extreme examples are the food pathogen *E. coli* EHEC O157:H7 strain Sakai carrying 18 prophages which amount to 16% of the total genome or *S. pyogenes* with up to six prophages comprising 12% of the bacterial genome [51,52]. Stable prophages that are typically observed in isolated bacterial cultures have a long-lasting bond with their hosts comparable to a symbiosis finally supporting duration of both. However, it is important to note that prophages can either cause loss of host fitness or, just the opposite by adding new functions to the cell via lysogenic conversion comparable to a “gain-of-function”. Still another type of host–phage interaction is active lysogeny: prophages may integrate in critical bacterial genes such that they function as regulatory switches [50]. Stable prophages tend to protect their bacterial hosts until “no other choice but escape” is left. In this context, it is important to distinguish precisely between different genetic transfer mechanisms like general and specialized transduction and lysogenic conversion. Bacteria may benefit from lysogenic conversion by obtaining novel virulence factors (toxins, super-antigens, immune evasion, invasivity, adherence, resistance to phages) [53,54]. It might be postulated that cryptic prophages and active lysogeny are part of bacterial regulatory systems. The interaction between such prophages and lytic phages could be considered as interaction between bacterial genomes and phages or, between lysogenic and lytic phages. Such questions are required to get in focus of fundamental research. For the purpose of this article, it is important to understand the biology of obligately lytic and of temperate phages. There are many fascinating and as yet unexplored aspects of the microbial and phage world, however, conditions outside the laboratory or production facility are not a priority for this article.

### 4.2. Detection of Prophages in Bacterial Genomes

There is consensus among experts that host strain genome sequencing is an essential initial investigation before starting phage production processes as it allows the identification of prophage genes like integrases, repressors, excisases, recombinases, terminases. Having this sequence available thus makes predictions for prophage properties like virulence factors or for prophage incompleteness possible. Induction ability might be experimentally studied by using Mitomycin C or UV irradiation but these induction methods are not successful enough and require indicator strains that are not always available. Due to the increasing importance of phage applications, entering prophage genomes in databases is necessary when bacterial genomes are analyzed. Algorithms for finding prophages are available like PhiSpy [55], PHAST (http://phast.wishartlab.com) and PHASTER [56] and genome annotations will shed light on prophage properties and this is getting less complicated the more sequence data are entered into databases. We strongly encourage researchers to make such data publically available.

### 4.3. Prophages in Bacterial Strains Used to Produce Therapeutic Phages

Finding production strains that are completely free of prophages is generally difficult, especially in the case of some pathogens. Therefore, inducibility and the genetic outfit of a prophage should be considered, definitions are necessary to deal with this issue. Genome sequencing is indispensable as prophages might be carriers of pathogenicity factors, as well as antibiotic and phage resistance determinants. Isolation of new phages aiming at therapeutic preparations cannot initially exclude temperate phages but further characterization steps should definitely eliminate them. Regarding the production strain and production process, the individual situation has to be assessed: is a strain available (and effective!) that is lacking prophages? If not, do bioinformatic genome analyses describe the prophage’s genetic outfit sufficiently to confirm a cryptic nature? Is there a potential of infectious particles being released into the medium during production and if so, how probable is this event? If all these evaluations conclude that the production strain does not present likelihood of a problem, the final point should be considered that in a special situation the preparation may be the only viable alternative left, this is especially the case for magistral purposes. It is also important to note that a phage production strain must efficiently produce phages, meaning the EOP has to be satisfactory. Expert consensus was that production strains should ideally not carry prophages or should no other suitable strain exist then the risk needs to be controlled to minimize prophage expression [35].

We suggest that the significance of this concern must be put into perspective and seen in comparison to the global multidrug resistance crisis and the human crisis in individual cases. Improvement of phage production is important but it is fortunate that (1) lytic phages for clinical isolates of the ESKAPE bacteria (see above) are not difficult to isolate and (2) a rather good number of production strains within the ESKAPE bacteria should be available. Careful monitoring of the production process and where possible, a pre-adaptation of the therapeutic phage candidates to suitable production strains is a way forward. Repeated phage genome sequencing during the production process seems mandatory. Gene modification of production strains is discussed, but may only be one way out in extremely challenging cases in the future whereas currently, experts agree that eliminating prophages from the bacterial genome should not become a prerequisite. Q-PCR or sequencing can confirm purity of the DNA of the lytic phage and exclude prophage DNA. According to one expert, one possible way to assess the capacity of prophage elements to successfully induce under production conditions involves the use of Q-RT-PCR to track the concentration of DNA for each prophage relative to other genomic DNA during infection with primers specific to each prophage element as was done by Ceyssens et al. [57]. If replication of prophage DNA relative to other genomic DNA does not occur, this would definitively exclude successful prophage induction.

As a conclusion, it is obvious that more phage research is required going hand-in-hand with applied therapy approaches since an easy solution to the “prophage problem” is not obvious. Indeed, this is even more problematic if a phage should be amplified on a freshly isolated patient strain (see magistral application [38]). If compared to a threshold endotoxin limit in phage preparations, it might be feasible to define such a limit for prophage presence. Mathematical modeling of phage–bacterium pharmacodynamics may allow simulation of whether such concentrations are significant in terms of propagation of the temperate phages; low concentration of temperate phages may bear a very low statistical probability of significant amplification as the phage binding process is concentration dependent. Such thoughts seem theoretical but might be key for the design of strategies in the near future and for negotiating with licensing authorities.

### 4.4. Prophages in Production Strains: What Does It Mean for the Licensing Pathway?

It must be stated that intact prophages are not necessarily induced and released at high frequency. If a production strain contains a frequently inducible intact prophage it should not bear antibiotic resistance genes, toxin genes or other virulence factors to reduce the risk of gene transfer. The presence of temperate phages in a therapeutic preparation should normally not affect its efficacy (see above). Lytic phages can in principal also contribute to horizontal gene transfer via generalized transduction, but a risk–benefit evaluation must be rational, and a pragmatic common-sense approach is needed; just look at the human microbiome and its virome where prophages are the most frequent inhabitants! For commercially available phage preparations in the future it should be compulsory that production strains are free of functional prophages whereas exceptions might be made for highly experimental treatments. Generally, functional-prophage-free production strains should be the primary choice, however, other parameters need consideration including determining the suitability of a production strain e.g., efficacy and prophage-independent pathogenicity factors etc. For regulatory pathways, some kind of “prophage acceptance threshold” should be defined, it would contribute to a constructive timely European approach to design the regulatory framework for safe phage therapy. Furthermore, the establishment of collections/banks of prophage-free potential production strains, especially those of the ESKAPE bacteria, is urgently needed. Such strains can sometimes be acquired by natural means, such as prophage induction and prophage-free cell selection, without the use of genetic modifications [58].

## 5. Concluding Remarks

### 5.1. Bacterial Phage Resistance

It is commonly accepted that combining phages with different infection strategies into cocktails will help reduce the selection of phage resistant bacterial clones, but this point has only been studied on a few occasions both in vitro and in vivo. Sequential strategies might be interesting when there is no sense of urgency (e.g., long-term phage therapy of chronic infections), but more research is needed here. In acute life-threatening infections, and in anticipation of rapid (automated) bacterial identification and phage selection and adaptation techniques, well-thought-out broad-spectrum phage cocktails are warranted. More in vivo studies are needed to document the impact of phage pre-adaptation on bacterial phage resistance reduction. More research is needed to determine if the theoretical bacterial phage resistance issue (if phage therapy would be applied intensively, which, even in Georgia, is not the case today) will be comparable to the persistent bacterial multidrug resistance problem we are facing today. Meanwhile, most experts suggest restricting the use of phages, to a greater or lesser extent, to limit the potential spread of bacterial phage resistance in anticipation of relevant data.

### 5.2. Phage Training

Phage training is an experimental co-evolution approach considerably accelerating the pace at which “increased” phages are selected, thanks to the short doubling time of many bacterial species. Ultimately, naturally pre-adapted phages could be very interesting alternatives to original phages if their improved abilities translate from the preclinical situation to the clinic. Therefore, we believe that trained phages should be included in products to be tested in clinical trials, provided that their safety and superior efficacy compared to the ancestral phage have been documented in in vitro pre-clinical studies. In addition to their potential increased efficacy, broad host and/or variant range of trained phages could offer a significant economic advantage regarding the costs of production in GMP, which is a pre-requisite to prospective clinical trials.

### 5.3. Prophages in Bacterial Production Strains

Temperate phages/prophages present a problem in phage production processes, but are so abundant in all habitats including our own microbiome that we should not blindly demand their elimination from phage production processes. Instead, we broadly advise that an intensive discussion be had on how to deal with situations when no prophage-free production strains are available. We have explained which properties prophages might carry and what their unwanted features are, how abundant they are in opportunistic common ESKAPE bacteria, how genome analyses precisely characterize them and what to calculate if a production strain carries a prophage. We advocate that researchers enter bacterial and (pro)phage genome data into public databases because rich databases will provide the platform that is needed to deal with these particular pressing questions and because phage therapy will be a potent alternative therapy in the global multidrug resistance threat. Resolute and concurrent activities are urgently needed and harmonized licensing pathways are desirable. They might include best practice guidance for those few gaps that cannot be completely solved when a biological therapy approach is used; this may include the prophage issue.
